# Cerium Oxide Nanoparticles Conjugated with Tannic Acid Prevent UVB-Induced Oxidative Stress in Fibroblasts: Evidence of a Promising Anti-Photodamage Agent

**DOI:** 10.3390/antiox12010190

**Published:** 2023-01-12

**Authors:** Regina G. Daré, Elayaraja Kolanthai, Craig J. Neal, Yifei Fu, Sudipta Seal, Celso V. Nakamura, Sueli O. S. Lautenschlager

**Affiliations:** 1 Post-Graduate Program in Pharmaceutical Sciences, State University of Maringá (UEM), Maringá 87020900, Paraná, Brazil; 2 Advanced Materials Processing and Analysis Center, Materials Science and Engineering, University of Central Florida, Orlando, FL 32816, USA; 3 Nanoscience Technology Center (NSTC), and Biionix Cluster, Department of Internal Medicine, College of Medicine, University of Central Florida, Orlando, FL 32816, USA; 4 Department of Basic Health Sciences, State University of Maringá (UEM), Maringá 87020900, Paraná, Brazil

**Keywords:** antioxidant, nanoceria, phenolic compound, photoaging, ultraviolet radiation

## Abstract

Exposure to ultraviolet radiation induces photodamage towards cellular macromolecules that can progress to photoaging and photocarcinogenesis. The topical administration of compounds that maintain the redox balance in cells presents an alternative approach to combat skin oxidative damage. Cerium oxide nanoparticles (CNPs) can act as antioxidants due to their enzyme-like activity. In addition, a recent study from our group has demonstrated the photoprotective potential of tannic acid (TA). Therefore, this work aimed to synthesize CNPs associated with TA (CNP-TA) and investigate its photoprotective activity in L929 fibroblasts exposed to UVB radiation. CNP conjugation with TA was confirmed by UV–Vis spectra and X-ray photoelectron spectroscopy. Bare CNPs and CNP-TA exhibited particle sizes of ~5 and ~10 nm, superoxide dismutase activity of 3724 and 2021 unit/mg, and a zeta potential of 23 and −19 mV, respectively. CNP-TA showed lower cytotoxicity than free TA and the capacity to reduce the oxidative stress caused by UVB; supported by the scavenging of reactive oxygen species, the prevention of endogenous antioxidant system depletion, and the reduction in oxidative damage in lipids and DNA. Additionally, CNP-TA improved cell proliferation and decreased TGF-β, metalloproteinase-1, and cyclooxygenase-2. Based on these results, CNP-TA shows therapeutic potential for protection against photodamage, decreasing molecular markers of photoaging and UVB-induced inflammation.

## 1. Introduction

Repetitive exposure of the skin to ultraviolet (UV) radiation causes an imbalance in the redox state of the cellular environment, leading to oxidative stress, the starting point of skin photodamage, which in turn provokes photoaging and photocarcinogenesis. Photoaging leads to profound changes in the composition and architecture of the dermal extracellular matrix [1]. Additionally, photocarcinogenesis involves multiple steps of biochemical reactions, including tumor initiation, tumor promotion, and tumor progression, eventually causing skin cancer [2]. Moreover, UV radiation leads to high-intensity inflammatory responses in acute exposure, causing sunburn, and in chronic exposure, leads to low-intensity inflammatory reactions, which are closely related to photoaging, immunosuppression, photocarcinogenesis and skin cancer development comprised of melanoma and non-melanoma types [3,4]. Among the possible mechanisms that explain the damage caused by UV radiation to the skin is the intense generation of reactive oxygen species (ROS), and the inability to neutralize them by the endogenous antioxidant defense mechanism, which leads to oxidative stress followed by direct oxidative damage to cellular macromolecules including lipids, proteins, and DNA [5]. As a long-term consequence, a variety of pathological processes are aggravated or caused by exposure to solar radiation [6,7].

The prevention and treatment of injuries induced by exposure to UV radiation are of great importance for promoting the health of populations that are more frequently exposed to solar radiation. In addition, it is known that life expectancy is longer compared to past decades [8]. As the damage caused by UV exposure is cumulative, increased longevity favors the emergence of photoinduced chronic changes, such as the intensification of photoaging and the increased incidence of skin cancers, especially in elderly populations, reflecting the long latency between exposure to the carcinogen and cancer formation [9]. The therapeutic modalities for the management of photodamage include the topical administration of sunscreens and antioxidants, which reduce the oxidative injuries of the integumentary tissue cells, compromising keratinocytes and fibroblasts. In this context, studies demonstrate the beneficial properties of phenolic compounds as preventive agents against photodamage in skin cells [10], combating oxidative damage to macromolecules and decreasing the prevalence of inflammatory processes [11].

A recent study of our research group evidenced that tannic acid (TA), a phenolic compound, exhibited high antioxidant capacity and that TA treatment in UVB-irradiated fibroblasts prevented cellular redox imbalance, oxidative damage to cellular macromolecules, and photoaging [12]. In another study from our group [13], the photoprotective potential of cerium oxide nanoparticles (CNPs) was evaluated. CNP treatment was able to combat cellular photodamage in UVB-irradiated fibroblasts by decreasing ROS generation and increasing the activities of endogenous antioxidant enzymes. There has been an increase in interest in the application of CNPs as antioxidants for a variety of treatments related to oxidative stress disorders, due to their mimetic antioxidant superoxide dismutase (SOD) and catalase (CAT) activity and their direct ROS scavenging effect in cell and animal models. The antioxidant effect of CNPs depends on their physicochemical properties, as their nanoscale size offers the thermodynamic efficiency of the redox cycling between cerium 3+ (Ce^3+^) and cerium 4+ (Ce^4+^) ions on the nanoparticle surface, and the ability to absorb and release oxygen [14].

To date, to the best of our knowledge, few studies have been reported the effects of cerium oxide nanoparticles against UV radiation-induced oxidative photodamage in cells. For instance, zholobak et al. [15] reported that CNPs prevented the decrease in cell viability of mouse fibroblasts (L929) and fibroblast-like cells of African Green monkey (VERO) exposed to UV. Fujita and Kamada [16] demonstrated that CNPs decreased oxidized DNA under UV light. Zholobak et al. [17] reported that panthenol-stabilized CNPs protect human epidermoid cancer cells (Hep-2) and the diploid epithelial swine testicular cell line (ST-cells) from ROS and UV irradiation. Additionally, Ribeiro et al. [18] showed that CNPs with a higher Ce^3+^ content decreased UVA-induced L929 fibroblast death by regulating protein kinases 1 and 2 (ERK 1/2). Therefore, the present study proposed to synthesize a novel delivery system of TA conjugated with CNPs (CNP-TA) and to investigate the photoprotective potential of this nanoparticulate system, using as in vitro model L929 fibroblasts exposed to UVB, with emphasis on combating photodamage processes.

## 2. Materials and Methods

### 2.1. Materials

All chemicals and solvents used were of analytical grade. Tannic acid (purity 95%) was purchased from ACROS organics (Geel, Belgium, Europe). Bradford reagent was acquired from Bio-Rad (Hercules, CA, USA). Acridine orange, catalase, cerium nitrate hexahydrate (purity 99.999%), dimethyl sulfoxide (DMSO), ethyl diamine, HBSS (Hank’s balanced salt solution), hydrogen peroxide (99.9%), ortho-phthalaldehyde, protease inhibitor cocktail, reduced glutathione (GSH), and the chemical cross-linkers of 1, 1′-carbonyldiimidazole (CDI) were obtained from Sigma-Aldrich (St. Louis, MO, USA). DPPP (diphenyl-1-pyrenylphosphine) and H_2_DCF-DA (2′,7′-dichlorodihydrofluorescein diacetate) were purchased from Molecular Probes (Eugene, OR, USA). DMEM (Dulbecco’s modified Eagle’s medium) and fetal bovine serum were purchased from Gibco (Carlsbad, CA, USA). Neutral red (3-amino-7-dimethylamino-2-methylphenazine hydrochloride) was obtained from Inlab (São Paulo, Brazil). HRP-conjugated goat anti-rabbit IgG secondary antibody, mouse anti-COX-2, mouse anti-MMP-1, mouse anti-β-actin and ECL reagent were purchased from Santa Cruz Biotechnology (Paso Robles, CA, USA).

### 2.2. Tannic Acid Conjugation with CNP

The cerium oxide nanoparticle was synthesized by a wet chemical method based on work published elsewhere [19]. Briefly, cerium nitrate hexahydrate (Ce(NO_3_)_3_·6H_2_O) and hydrogen peroxide (3 *v*/*v* %) were used as reactants for nanoparticle synthesis. Initial preparation involved dissolving cerium nitrate hexahydrate in deionized water, followed by adding hydrogen peroxide to the cerium nitrate solution and mixing at room temperature. In combination with hydrogen peroxide, cerium ions are oxidized, resulting in a yellow solution, which becomes clear as particles undergo condensation and solution dispersion. The oxidation process occurs at room temperature and fully aged nanoparticles are ready for use after 6 to 8 weeks.

**Figure 1:**
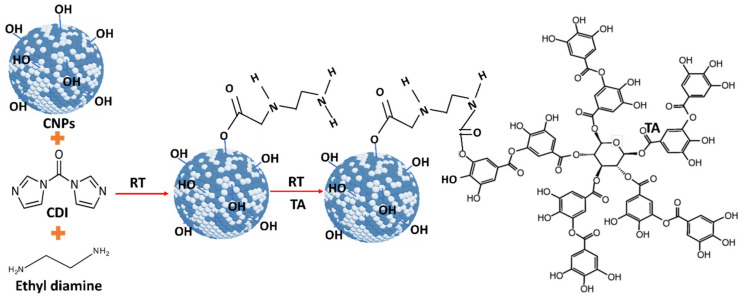
Schematic diagram representing the cerium oxide (CNPs) and tannic acid (TA) conjugation by imidazole chemistry at room temperature. Initially, the hydroxyl group (OH) on the CNP surface was activated by 1, 1′-carbonyl diimidazole (CDI) followed by ethylene diamine (EDI) added to the activated CNP solution. One end of the amine group EDI is attached to the active imidazole carbamate intermediate, and another end is attached to a hydroxyl group in TA molecules to form two carbamide bonds to conjugate TA and CNPs.

As-synthesized water-dispersed-cerium oxide nanoparticles (CNPs) were used for conjugation. Initially, 100 μL of synthesized 5 mM CNPs was diluted into 900 μL of DMSO solvent. 100 μL of CDI (500 mM) solution was added to CNPs to activate the OH group on CNPs. This solution was continuously shaken for 1 h using shake-plate equipment, at room temperature. After activation of the OH group, 250 μL of ethyl diamine was added to activated CNPs, and it was continuously shaken for another 2 h. Then, 225 µL of 0.5 mg/mL tannic acid solution was added to the CNPs solution and it was continuously stirred overnight to conjugate the tannic acid with CNPs. In ethyl diamine, one end of the amine group is attached to the CDI-CNPs [20], and another end is attached to the OH group in the TA molecule (Figure 1). Any un-conjugated nanoparticles and drug molecules were removed by the dialysis process performed for 24 h against RNAase-free water. The tannic acid-conjugated CNPs (CNP-TA) samples were stored at −4 °C up to further use.

### 2.3. CNP-TA Physicochemical Characterization

Before and after TA conjugation with CNPs, samples were subjected to various characterizations. UV–Vis spectra of samples were analyzed. The surface properties of the oxidation state of pure and TA-conjugated CNPs were examined by 5400 PHI ESCA X-ray photoelectron spectroscopy (XPS) (RBD Instruments Inc., Bend, OR, USA) with Mg-Kα X-radiation (1253.6 eV) at 350 W and pressure 10^−9^ Torr maintained during the measurement. C-1S peak at 284.6 eV was used as a reference to compensate for any peak shift due to charging. The size and morphology of the drug-conjugated samples were analyzed by 300 keV high-resolution transmission electron microscopy (HR-TEM) with an FEI Tecnai F30 EDX analyzer (Jeol Inc., Peabody, MA, USA). Diluted pure and drug-conjugated samples drop cast on a carbon-coated 400 mesh copper grid and dried under the IR lamp. After, SOD activity was analyzed for the pure and drug-conjugated CNPs samples. The SOD activity was tested on pure, drug-conjugated CNPs and the positive kit control using the Dojindo SOD assay kit (Rockville, MD, USA). The different concentrations of pure and drug-conjugated CNPs were prepared using deionized water and examined for SOD activity. The absorbance at 450 nm was measured while xanthine oxide and samples interacted in the presence of WST-1 dye. Linear fitting was applied to these data, and the slope value was determined. The percentage of SOD activity for conjugated samples was calculated from the estimated slope value and compared against SOD control data. The experiment was performed in triplicate in each condition of the samples. Further, the zeta potential of TA-conjugated CNPs was analyzed using a Nano-ZS zeta sizer (Malvern Instruments, Swavesey, Cambridge, UK) instrument. Additionally, the conjugated samples were subjected to inductively coupled plasma mass spectrometry (ICP-MS) analysis to determine the concentration of cerium. The fixed volume of drug-conjugated samples was digested in 35% nitric acid solution and incubated for 48 h at 80 °C in a conventional hot air oven. The digested sample was diluted further with deionized water to maintain a 5% nitric acid concentration in the final solution. To quantify the cerium concentration in the synthesized nanoparticles, the Agilent 7700X (Agilent Technologies Inc., Santa Clara, CA, USA) inductively coupled plasma-mass spectrometry facility was used at Florida International University. Bond character and chemistry for TA, CNP and CNP-TA samples were analyzed using Fourier-Transformation Infrared Spectroscopy (FTIR). IR spectra were recorded in the range of 4000–500 cm^−1^, with a Perkin-Elmer *Spectrum One* FTIR (Waltham, MA, USA) by dispersing in KBr.

### 2.4. Cell Culture and UVB Irradiation

Photoprotection assessment of free TA, CNPs, and CNP-TA was evaluated in L929 fibroblasts (ATCC CCL1™, Manassas, VA, USA). Cells were cultured in DMEM supplemented with 10% (*v*/*v*) fetal bovine serum, penicillin (100 U/mL), and streptomycin (100 µg/mL), maintained at 37 °C under 5% CO_2_ atmosphere. For the irradiation process, the culture medium was replaced by HBSS buffer. Then, the cells were exposed to UVB (UVB lamp: TL40 W/12 RS; Philips; 290–315 nm; peak intensity 312 nm) at an intensity of 500 mJ/cm^2^, monitored using a radiometer (VLX-3W, Vilber Lourmat, Eberhardzell, Baden-Wurttemberg, Germany).

### 2.5. Cell Viability

To evaluate the effects of free TA, CNPs, and CNP-TA on the viability of L929 fibroblasts, cells grown in 96-well plates (2.5 × 10^5^ cells/mL) were treated with different concentrations of the samples (5, 10, 20, 50, 100 and 200 µg/mL) for 24 h. For the evaluation of cell viability in fibroblasts treated and irradiated, cells cultivated in 24-well plates (2.5 × 10^5^ cells/mL) were treated for 1 h or 24 h with different concentrations of the samples (5, 10, 20, 50, and 100 µg/mL), exposed to UVB and incubated for 24 h. After the incubation period of the respective experiments, the neutral red assay was performed to assess cell viability [21]. The cells were incubated for 3 h with neutral red (40 µg/mL), fixed with formaldehyde (2%) and CaCl_2_ (1%) for 5 min, and the neutral red retained in the cells was solubilized with 50% ethanol/1% acetic acid. Measurements were taken at 540 nm (Bio-Tek^®^, Power Wave XS, Hampton, VA, USA).

### 2.6. Reactive Oxygen Species Detection

To evaluate ROS generation L929 fibroblasts cultured in 96-well plates (2.5 × 10^5^ cells/mL) were treated for 24 h with free TA, CNPs, and CNP-TA (5, 10, 20, 50, and 100 µg/mL), then incubated with H_2_DCF-DA (10 µM) for 45 min at 37 °C. Then, the cells were exposed to UVB, and measurements were immediately taken. Fluorescence was detected at 488/525 nm excitation/emission (Victor^®^ X3, Perkin Elmer, Waltham, MA, USA).

### 2.7. Endogenous Antioxidant Defense System Evaluation

The endogenous antioxidant defense system was analyzed by evaluating the enzymatic activity of CAT and the levels of glutathione (GSH). Briefly, L929 fibroblasts were cultured in 6-well plates (4 × 10^5^ cells/mL), treated for 24 h with free TA, CNPs, and CNP-TA (10 and 20 µg/mL), exposed to UVB and incubated for 24 h. Afterward, cell lysates were prepared by scraping the cells in lysis buffer [10 mM Tris-HCl (pH 7.4) and 1% triton X-100], followed by sonication for 60 s and centrifugation at 10,000× *g*/10 min/4 °C. The supernatants were collected, and the amount of protein was estimated [22]. For CAT activity [23], potassium phosphate buffer (50 mM, pH = 7.0), cell lysate (equivalent to 50 μg of protein), and H_2_O_2_ (30 mM) were added to a quartz cuvette. The reaction was monitored at 240 nm for 5 min at 25 °C (Shimadzu, UV-1700, Columbus, GA, USA), and the results were expressed according to the calibration curve of the bovine liver CAT standard (E.C. 1.11.1.6) (0.391–50 U/mL). For the GSH assay [24], in a 96-well black plate were added 180 μL of sodium phosphate buffer (100 mM, 5 mM EDTA, pH 8.0), 10 μL of cell lysate and 10 μL of ortho-phthalaldehyde. Fluorescence was detected after 15 min of incubation at room temperature at 350/420 nm excitation/emission (Victor^®^ X3, Perkin Elmer, Waltham, MA, USA), and the results were expressed according to the GSH standard calibration curve (1.953–1000 μg/mL).

### 2.8. Cellular Photodamage Evaluation

To assess lipid peroxidation, L929 fibroblasts cultured in 96-well plates (2.5 × 10^5^ cells/mL) were treated for 24 h with free TA, CNPs and CNP-TA (10 µg/mL), exposed to UVB and incubated for more 24 h. Then, the cells were incubated with DPPP (20 μM) for 30 min at 37 °C. The fluorescence was determined immediately after at 351/460 nm excitation/emission (Victor^®^ X3, PerkinElmer, Waltham, MA, USA) and the amount of protein was estimated [22].

To assess DNA damage, DNA condensation and fragmentation were evaluated by acridine orange staining and agarose gel electrophoresis, respectively. For DNA condensation analysis, L929 fibroblasts cultured in 24-well plates under glass coverslips (2.5 × 10^5^ cells/mL) were treated for 24 h with free TA, CNPs and CNP-TA (10 µg/mL), exposed to UVB and incubated for another 24 h. Then, the cells were stained with acridine orange (1 μg/mL), incubated at room temperature for 10 min, and the images were recorded under a fluorescence microscope (Olympus^®^, BX51, Olympus America Inc., Melville, NY, USA). The images were also analyzed quantitatively by the ImageJ software version 1.51 (National Institutes of Health, Bethesda, MD, USA). For the analysis of DNA fragmentation, L929 fibroblasts cultured in 6-well plates (4 × 10^5^ cells/mL) were treated for 24 h with free TA, CNPs and CNP-TA (10 µg/mL), exposed to UVB and incubated for another 24 h. DNA isolation and electrophoresis process were carried out as described by [12], using a modified methodology proposed by [25]. The DNA isolates were subjected to electrophoresis in a 0.7% agarose gel containing SYBR^®^ Safe DNA dye for 1 h at 80 V. The images of the obtained gels were recorded using the GelDoc^®^ XR+ Imaging System (Bio-Rad Laboratories Inc., Hercules, CA, USA).

### 2.9. Wound Healing Scratch Assay

To assess cell proliferation after UVB exposure, a sterile 200-µL tip was used to make a straight scratch on the monolayer of L929 fibroblasts plated in 24-well plates (2.5 × 10^5^ cells/mL). Then, the cells were treated for 24 h with free TA, CNPs and CNP-TA (10 µg/mL) and exposed to UVB. Then, DMEM containing 0.5% fetal bovine serum was added to the wells, and pictures were taken immediately after irradiation (0 h) and 24 h after irradiation. Wound repopulation was visualized under an optical microscope (Olympus^®^, CKX31, Olympus America Inc., Melville, NY, USA) and images were captured at 5× magnification. The wound repopulation area was measured using ImageJ software version 1.51 with a wound healing plugin.

### 2.10. TGF-β Levels Detection

To assess TGF-β levels, L929 fibroblasts cultured in 6-well plates (4 × 10^5^ cells/mL) were treated for 24 h with free TA, CNPs and CNP-TA (10 µg/mL), exposed to UVB and incubated for another 24 h. Then, the supernatants were used for TGF-β1 quantification using an ELISA kit (enzyme-linked immunosorbent assay), according to the manufacturer’s instructions (88–8350, ThermoFisher, Vienna, Austria). Recombinant human/mouse TGF-β was used as standard for the calibration curve.

### 2.11. Western Blot

L929 fibroblasts cultured in 6-well plates (4 × 10^5^ cells/mL) were treated for 24 h with free TA, CNPs and CNP-TA (10 µg/mL), exposed to UVB and incubated for another 24 h. Afterward, cell lysates were prepared with lysis buffer [150 mM sodium chloride, 5 mM EDTA, 50 mM tris-HCl (pH 8.0), 1% triton X-100, 5% dodecyl sulfate sodium and 1% protease inhibitor cocktail]. Cell lysates were further sonicated for 2 min (60% amplitude), centrifuged at 10,000× *g*/20 min/4 °C and the amount of protein was estimated [22]. Equal amounts of cell lysates were diluted in sample buffer [5% mercaptoethanol, 5% bromophenol blue, 75 mM tris-HCl (pH 6.8), 2% sodium dodecyl sulfate and 10% glycerol], heated to 100 °C for 5 min and subjected to 12% sodium dodecyl sulfate-polyacrylamide gel electrophoresis. Then, the proteins were transferred to a nitrocellulose membrane (GE-Healthcare, Germany) in transfer buffer (25 mM tris, 192 mM glycine, 20% methanol and 0.01% sodium dodecyl sulfate). Membranes were blocked with 5% albumin in TBS-T buffer [25 mM Tris-HCl (pH 7.4), 150 mM NaCl and 0.1% tween 20] for 1 h and incubated overnight at 4 °C with the anti-MMP-1, anti-COX-2 and anti-β-actin primary mouse antibodies. Then, the membranes were incubated for 1 h with HRP-conjugated goat anti-rabbit IgG secondary antibody. The ECL reagent was used to detect the antigen-antibody complex formed. Images were recorded using the ChemiDoc^®^ XRS+ Imaging System (Bio-Rad Laboratories Inc., Hercules, CA, USA).

### 2.12. Statistical Analysis

Results were expressed as the mean ± standard deviation of triplicates from three independent experiments. Data were analyzed on GraphPad Prism^®^ 5 using one-way ANOVA for different groups followed by Tukey’s test. Values of *p* < 0.05 were accepted as statistically significant.

## 3. Results

### 3.1. CNP-TA Physicochemical Characterization

**Figure 2:**
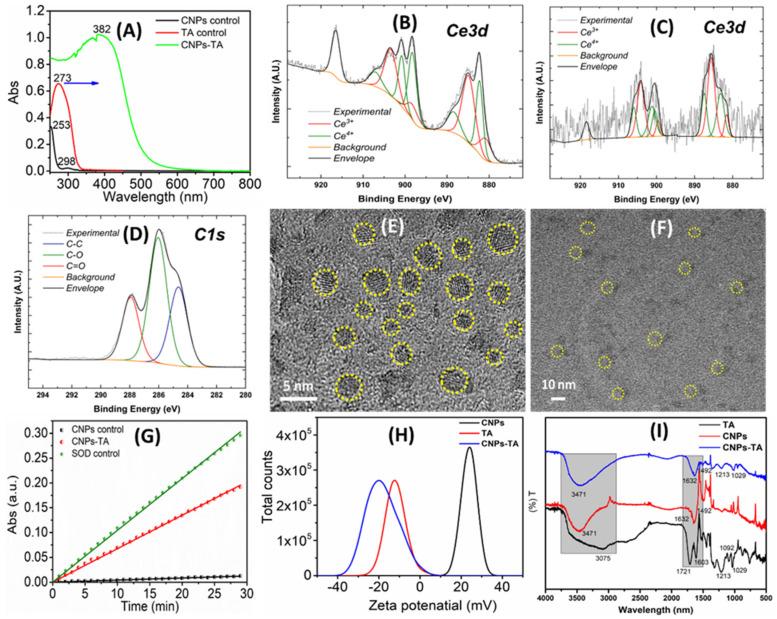
Shows various materials characterization techniques subjected to tannic acid (TA) molecules conjugated with cerium oxide nanoparticles (CNPs) by the bioconjugation process at room temperature. (**A**) UV−Vis spectrum of control CNPs (peaks at 253 and 298 nm), TA (peak at 273 nm), and CNPs−TA (peak at 382 nm). (**B**–**D**) X−ray photoelectron spectroscopy (XPS) spectrum of control CNPs, TA, and CNPs−TA. (**E**,**F**) High-resolution transmission electron microscopy (HR−TEM) images of control CNPs and CNPs−TA. (**G**) Superoxide dismutase (SOD) activity of CNPs−TA compared with control SOD and CNPs. (**H**) The zeta potential of pure and conjugated samples. The zeta potential of control CNPs showed 23 ± 2 mV, which changed to −19 ± 2 mV after conjugation of the TA molecule. (**I**) FTIR spectrum of TA, CNPs and CNP-TA samples.

The nanoparticles were subjected to various characterizations to confirm the CNP-TA conjugation, as well as discern its overall character (Figure 2). UV–Vis spectra of TA, CNPs, and CNP-TA are shown in Figure 2A. The water-based CNP sample showed two major absorbance peaks at 253 and 298 nm in the UV–Vis spectrum. These peaks correspond to the Ce^3+^ and Ce^4+^, respectively, redox states in the cerium oxide crystals. These results suggest the formation of cerium oxide particles synthesized by the wet-chemical hydrolysis method. An absorbance peak at 273 nm was observed for tannic acid samples, with this peak ascribed to a π → π* transition in the aromatic rings of the molecule [26]. For CNP-TA, a large broad peak was observed between 250 and 500 nm. Further, TA loading on CNPs was calculated from a standard concentration curve (linear fitting of known TA concentrations to absorbance values and extrapolation for measured sample values). The concentration of TA in CNP-conjugated samples is 64.9 µg/mL and this value is calculated from the standard curve.

XPS was performed to identify the distribution of cerium redox states in the material, as well as to demonstrate the presence of TA. The XPS of the control CNPs, TA, and CNP-TA are shown in Figure 2B–D, respectively. Ce 3d spectra (Figure 2B,C) demonstrate the mixed cerium valence with Ce^3+^ and Ce^4+^ states noted by fitted peak color. For the control CNP sample (Figure 2B), the ratio of Ce3+/Ce4+ was calculated as ~1.67 (64% Ce^3+^ and 36% Ce^4+^) from the sum fitted peak intensities specific to each redox state. The TA-conjugated sample (Figure 2C) similarly showed a mixed valence state, although with the redox state ratio decreased to 1.06 (51.36% Ce^3+^). Finally, a carbon (C1s) spectrum is shown (Figure 2D) with peaks appropriate for TA and without additional.

The size and morphology of control CNPs and CNP-TA were analyzed by HR-TEM (Figure 2E,F). The control CNPs showed spherical particles with sizes of 3 to 6 nm. After TA-conjugated CNPs, the particle size increased to approximately 10 nm without significantly altering the morphology. We should note that the image focusing on conjugated nanoparticles was impeded by the coating of organic (TA) molecules on the inorganic nanoparticles. Further, we analyzed whether SOD enzymatic activity is altered by the TA conjugation using a commercial Dojindo SOD kit (Figure 2G). The SOD activity of control CNPs and CNP-TA samples is 3724 and 2021 unit/mg, respectively. The zeta potential of control CNPs, TA, and CNP-TA was analyzed. The control CNPs showed a value of 23 ± 2 mV, which changed to −19 ± 2 mV after conjugation of the TA molecule (Figure 2H). In addition, the cerium concentration of CNPs-TA was quantified using the ICP-MS technique. The CNPs concentration in the conjugated sample was 144 ± 4 ppb (parts per billion).

Further, FTIR spectra of pure and TA-conjugated CNP samples were evaluated (Figure 2I). The absorption peaks centered at 1071 and 1092 cm^−1^ were assigned to C-H bending and aryl-oxygen stretching vibration modes of the TA molecule. The peak observed at 1213, 1309 and 1710 cm^−1^ were ascribed to O-H, C-O and C=O stretching vibration modes of TA. The broad absorption band between 3600 and 2500 cm^−1^ was assigned to O-H stretching vibration modes in TA [27]. The CNP sample showed peaks centered at 3471, 1632 and 1492 cm^−1^ ascribed to O-H stretching and bending vibration modes of surface species on the nanoparticles. Thereby, the presence of hydroxyl groups on CNP surface was confirmed by this analysis. CNP-TA sample measurements showed narrowed peaks of significantly reduced intensity for vibration modes assigned to hydroxyl groups, as compared to the control TA sample. The C=O stretching band related to the carbonyl group at 1721 cm^−1^ disappeared and a broader peak was observed at 1632 cm^−1^ for CNP-TA. This result clearly suggested the formation of amide bonding between CNP surface and TA molecules. Further, vibration peaks at 1213 and 1029 cm^−1^, unique to the TA molecules, were also observed in the CNP-TA sample. This is further evidence of the conjugation between CNPs and TA molecules.

### 3.2. CNP-TA Prevents UVB-Induced Cytotoxicity

**Figure 3:**
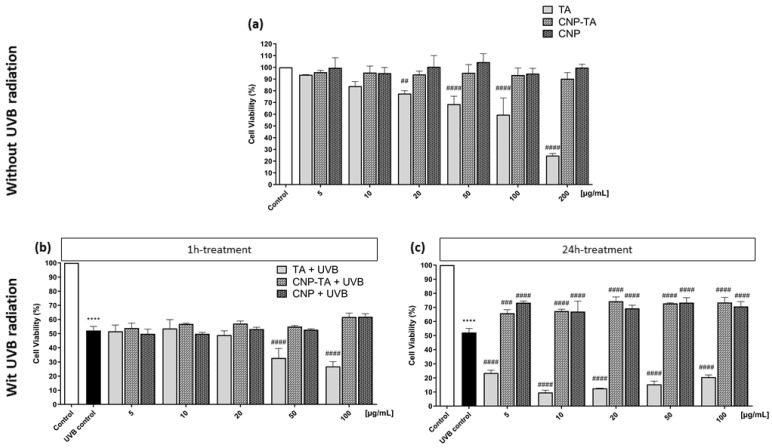
(**a**) Cytotoxicity effect evaluation of L-929 fibroblasts 24 h treated with TA, CNP-TA and CNPs, without UVB exposure. Control: untreated cells. ## *p* < 0.01 and #### *p* < 0.0001 compared to the control. (**b**,**c**) Effect of TA, CNP-TA and CNPs on cell viability in L-929 fibroblasts treated (5, 10, 20, 50 and 100 µg/mL) and exposed to UVB radiation. (**b**) Cells were treated for 1 h, irradiated and incubated for 24 h; (**c**) Cells were treated for 24 h, irradiated and incubated for an additional 24 h. Control: non-irradiated and untreated cells; UVB control: irradiated and untreated cells. **** *p* < 0.0001, compared to control, ### *p* < 0.001 and #### *p* < 0.0001 compared to UVB control.

Cell viability of L929 fibroblasts treated with TA, CNP-TA and CNPs was evaluated using a neutral red assay. First, the cytotoxic effect of different concentrations of samples was analyzed in cells treated for 24 h (Figure 3a). Treatment for 24 h with CNP-TA and CNPs did not decrease cell viability at all concentrations tested (5 to 200 µg/mL). However, free TA exhibited cell viability decrease in a dose-dependent manner from the concentration of 20 µg/mL (20 µg/mL: 22.4%; 50 µg/mL: 31.4%; 100 µg/mL: 40.4%; 200 µg/mL: 75.2% decrease in cell viability).

**Figure 4:**
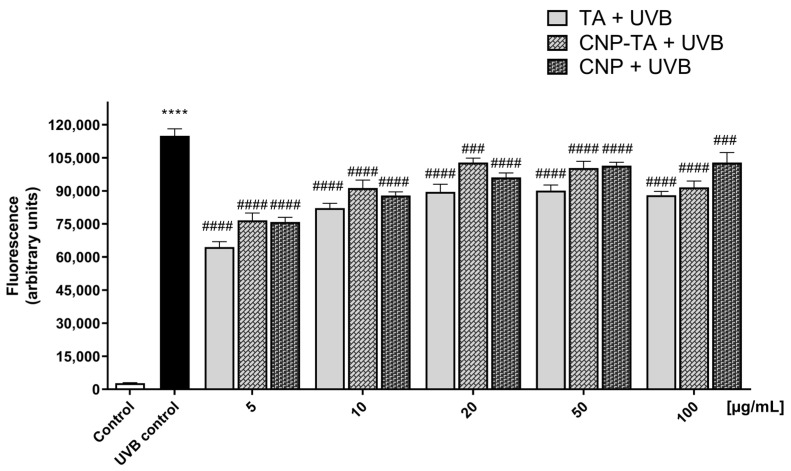
Evaluation of TA, CNP-TA and CNP treatments in the inhibition of reactive oxygen species (ROS) generation in L-929 fibroblasts. After 24 h treatment (5, 10, 20, 50 and 100 µg/mL), cells were exposed to UVB, and ROS were detected immediately after irradiation. **** *p* < 0.0001 compared to the control, ### *p* < 0.001 and #### *p* < 0.0001 compared to the UVB control. Control: non-irradiated and untreated cells; UVB control: irradiated and untreated cells.

Subsequently, the cytoprotection effect was evaluated for different concentrations of samples (5, 10, 20, 50, 100 µg/mL) in cells exposed to UVB radiation (Figure 4). Two treatment times were assessed: 1 h treatment (Figure 3b) and 24 h treatment (Figure 3c). Untreated and UVB-irradiated cells (UVB control) showed a 48% decrease in cell viability compared to untreated and non-irradiated cells (control). After 1 h of treatment, the samples were not able to prevent the decrease in cell viability in UVB-irradiated fibroblasts. However, for 24 h treatment, CNP-TA was able to significantly prevent the decrease in cell viability in irradiated cells, at all tested concentrations, with similar results (approximately 10% prevention of cell viability). The same was observed with the treatment of bare CNPs, in all concentrations tested. It is noteworthy that higher concentrations of free TA for 1 h treatment, and all concentrations for 24 h treatment, exerted a greater decrease in cell viability compared to UVB control. An effect that was not observed with CNP-TA, possibly indicating that when tannic acid is associated with CNPs, there is a decrease in the cytotoxic effect.

Based on these results, the 24 h treatment was chosen to proceed with further experiments, since this was the condition in which there was cytoprotection when cells were treated with CNP-TA and exposed to UVB.

### 3.3. CNP-TA Exhibits Protective Effect against UVB-Induced Oxidative Stress

To assess oxidative stress from ROS generation, activation of the endogenous antioxidant defense system (CAT and GSH) and oxidative damage to lipids and DNA were assessed. ROS generation was quantified using H_2_DCF-DA probe (Figure 4). The UVB control showed intense ROS production (4013% of increase), compared to the control. However, ROS production was significantly inhibited in cells treated and exposed to UVB radiation. For all samples, the lowest concentrations showed greater ROS inhibition (TA 5–100 µg/mL: 43–23%; CNP-TA 5–100 µg/mL: 38–3.8%; CNPs 5–100 µg/mL: 40–7% of ROS decrease). Based on these results, concentrations of 10 and 20 µg/mL of the samples were used to perform the subsequent experiment.

**Figure 5:**
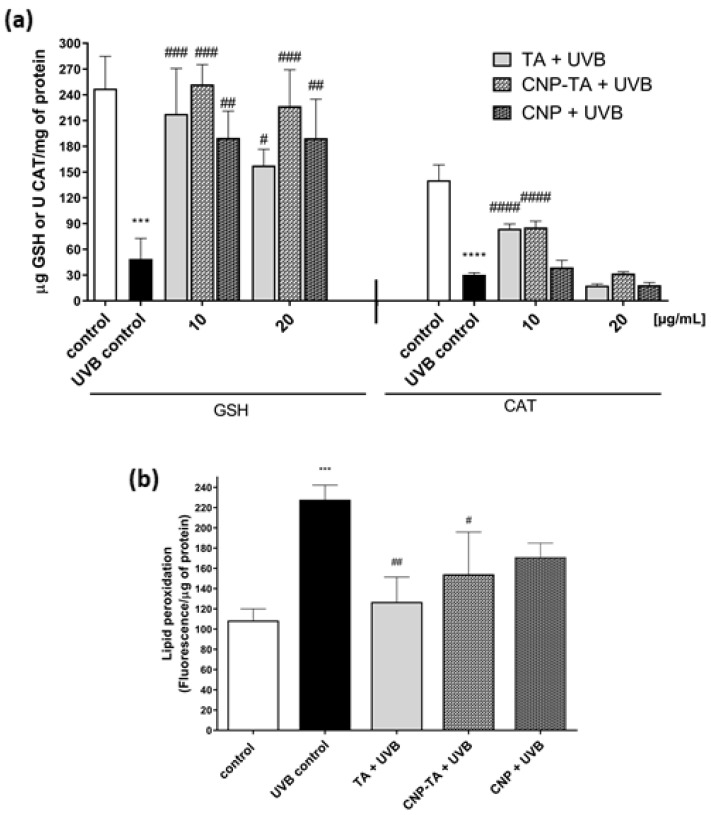
(**a**) Effect of TA, CNP-TA and CNPs on the restoration of reduced glutathione (GSH) and catalase enzyme (CAT) in L-929 fibroblasts treated (10 and 20 µg/mL) and irradiated with UVB. (**b**) Effect of TA, CNP-TA and CNPs against lipid peroxidation in L-929 fibroblasts treated (10 µg/mL) and irradiated with UVB. After 24 h of incubation, lipid peroxidation was measured using the DPPP marker. Control: non-irradiated and untreated cells; UVB control: irradiated and untreated cells. *** *p* < 0.001 and **** *p* < 0.0001 compared to the control, # *p* < 0.05, ## *p* < 0.01, ### *p* < 0.001 and #### *p* < 0.0001 compared to UVB control.

The levels of the antioxidant defense system were evaluated through the quantification of GSH and CAT (Figure 5a). The irradiated cells significantly decreased GSH levels and CAT activity by 79.7% and 78.4%, respectively, compared to the respective controls. When the cells were treated with the samples before being exposed to irradiation, there was prevention of depletion of the endogenous antioxidant system. For the GSH assay, CNP-TA and free TA showed better activity at the lowest concentration tested (TA 10 µg/mL: 346%; CNP-TA 10 µg/mL: 361%; CNPs 10 µg/mL: 288% increase in GSH levels). Additionally, for the CAT assay, only the lowest tested concentration of CNP-TA and free TA exhibited activity (TA 10 µg/mL: 176.7%; CNP-TA 10 µg/mL: 181.5% increase in the activity of CAT). Based on these results, a concentration of 10 µg/mL of the samples was used to perform further experiments.

Oxidative damage to lipids was analyzed using DPPP probe (Figure 5b). UVB radiation increased lipid peroxidation by 110% compared to the control. However, treatments with CNP-TA (32.3%) and free TA (44.3%) were able to significantly prevent UVB-induced lipid damage compared to UVB control.

**Figure 6:**
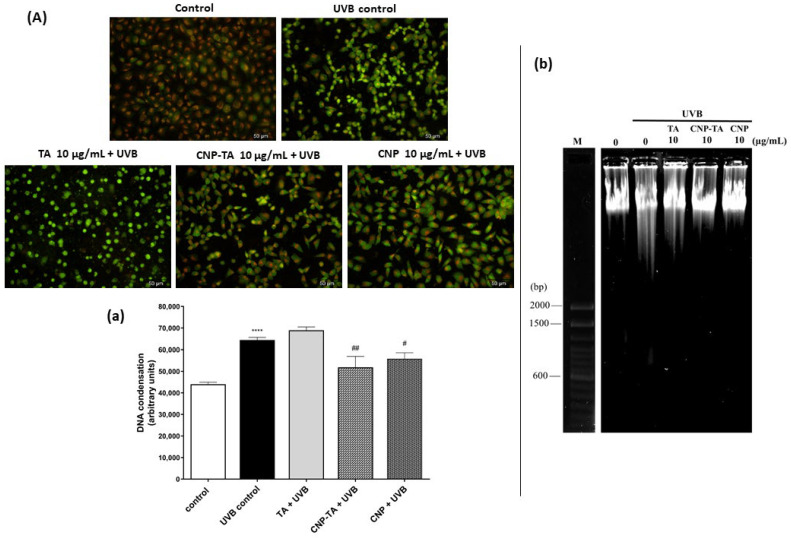
Effect of TA, CNP-TA and CNPs on DNA protection in L-929 fibroblasts treated (10 µg/mL) and irradiated with UVB. (**A**) Acridine orange staining: Cells were photographed using fluorescence microscopy (scale bar: 50 µm). The images are representative of three independent experiments: (**a**) five random images from each experiment were quantified using ImageJ software version 1.51. Control: non-irradiated and untreated cells; UVB control: irradiated and untreated cells. **** *p* < 0.0001 compared to control, # *p* < 0.05 and ## *p* < 0.01 compared to UVB control. (**b**) Agarose gel electrophoresis. M. DNA molecular weight marker (bp, base pairs; 100 base pair DNA ladder; Invitrogen, Waltham, MA, USA). Three experiments were carried out with similar results.

The evaluation of oxidative damage to DNA was evaluated according to its condensation and fragmentation intensity. To assess DNA condensation, cells were stained with acridine orange. Under this dye, the presence of bright green fluorescent spots indicates chromatin condensation. Figure 6A shows representative photomicrographs of three independent experiments and Figure 6a indicates the quantification of DNA condensation by ImageJ software version 1.51. The irradiated-only cells exhibited intense chromatin condensation, due to the intense green fluorescence of the cell nucleus (46.3% increase), in contrast to the non-irradiated cells, which did not exhibit intense green nuclear staining. Treatment with free TA, at a concentration of 10 µg/mL, was not able to reduce the extent of DNA condensation, but treatment with CNP-TA, at the same concentration, was able to reduce DNA condensation, with a reduction in green markings (19.4% reduction) compared to UVB control.

For the DNA fragmentation assay, the isolated DNA was subjected to agarose gel electrophoresis (Figure 6b). In the UVB-irradiated group, the DNA band was fragmented showing a smear pattern, in contrast to the untreated and non-irradiated (control) cells, which showed an intact DNA band. However, the treatment with the samples, mainly with CNP-TA and CNPs, avoided DNA fragmentation in the irradiated cells, as observed by the decrease in the drag of the DNA band along the gel.

### 3.4. CNP-TA Exhibits Potential in Attenuating Photodamage

**Figure 7:**
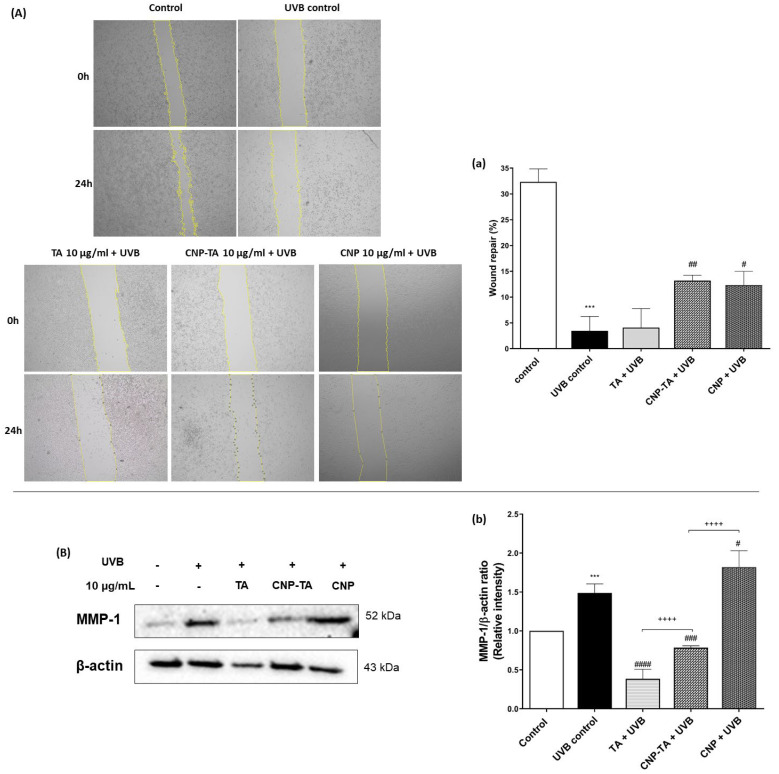
Effect of TA, CNP-TA and CNPs on photoaging prevention. (**A**;**a**) Effect of sample on wound repopulation in treated (10 µg/mL) and UVB-irradiated cells. (**A**) Cells were photographed by light microscopy (5× magnification). The images are representative of three independent experiments: (**a**) the wound (scratch) area was measured at 0 and 24 h using ImageJ software version 1.51. (**B**;**b**) Effect of samples on metalloproteinase-1 (MMP-1) protein expression in treated (10 µg/mL) and UVB-irradiated cells. Protein levels were normalized with β-actin. (**B**) Protein bands were recorded using ChemiDoc^®^ XRS+ Imaging System; (**b**) Band quantification was measured using ChemiDoc^®^ software version 2.0 (Bio-Rad, Hercules, CA, USA). Control: non-irradiated and untreated cells; UVB control: irradiated and untreated cells. *** *p* < 0.001 compared to the control, # *p* < 0.05, ## *p* < 0.01, ### *p* < 0.001 and #### *p* < 0.0001 compared to the UVB control, ++++ *p* < 0.0001.

Stimulation of cell proliferation was analyzed using the wound repair assay (Figure 7A,a). Wound recovery in cells under normal conditions (control) was 32% at 24 h compared to time zero. However, in irradiated and untreated cells (UVB control), cell proliferation of only 3.5% was observed in 24 h, compared to time zero, indicating the action of UVB radiation in preventing healing and tissue repair. In the cells treated with CNP-TA and CNPs, stimulation of cell proliferation after UVB exposure was observed (280% and 255% increase in wound repair, respectively). However, free TA showed no activity. In addition, the expression of metalloproteinase-1 (MMP-1) was analyzed by Western blotting technique (Figure 7b). UVB-irradiated cells exhibited a significant increase in MMP-1 (49%) compared to the control. However, treatment with both CNP-TA and free TA efficiently reduced MMP-1 levels by 47% and 74%, respectively, in irradiated cells compared to UVB control.

**Figure 8:**
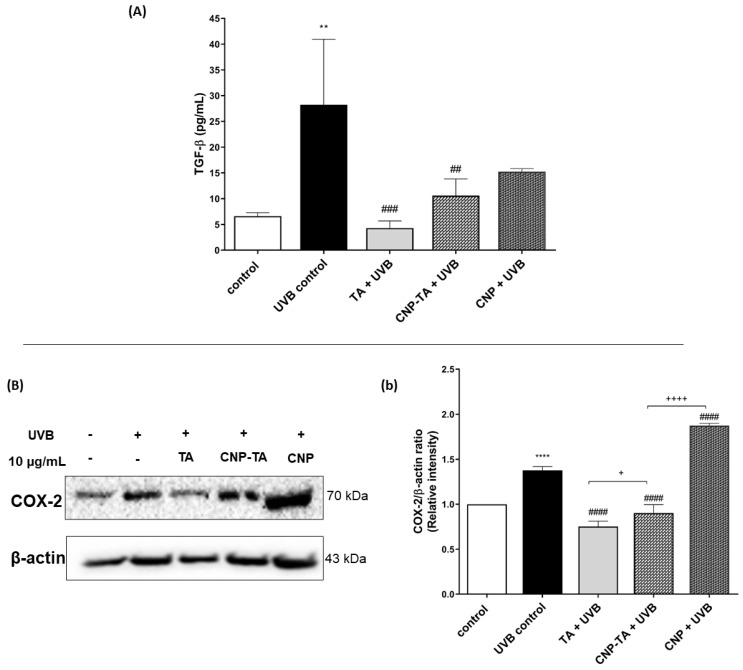
Effect of TA, CNP-TA and CNPs on the prevention of UVB-induced inflammation. (**A**) Effect of samples on the inhibition of transforming growth factor beta (TGF-β) in treated (10 µg/mL) and UVB-irradiated cells. (**B**;**b**)Effect of samples on cycloxygenase-2 (COX-2) protein expression in treated (10 µg/mL) and UVB-irradiated cells. Protein levels were normalized with β-actin. (**B**) Protein bands were recorded using ChemiDoc^®^ XRS+ Imaging System; (**b**) Band quantification was measured using ChemiDoc^®^ software version 2.0. Control: non-irradiated and untreated cells; UVB control: irradiated and untreated cells. ** *p* < 0.01, **** *p* < 0.0001 compared to control, ## *p* < 0.01, ### *p* < 0.001 and #### *p* < 0.0001 compared to UVB control, + *p* < 0.05 and ++++ *p* < 0.0001.

The assessment of CNP-TA potential to prevent UVB-induced inflammation was analyzed through the ability of the nanoparticles to inhibit the release of transforming growth factor beta (TGF-β) and to inhibit the expression of cycloxygenase-2 (COX-2) (Figure 8). TGF-β was quantified by the ELISA method (Figure 8A). UVB-irradiated cells exhibited a significant increase in TGF-β (327%) compared to the control. However, treatments with CNP-TA and free TA were able to reduce the levels of this cytokine by 62.4% and 84.8%, respectively, in irradiated cells compared to UVB control. CNPs showed no activity at the concentration tested. In addition, the expression of COX-2 was analyzed by Western blotting technique (Figure 8b). UVB-irradiated cells exhibited a significant increase in COX-2 (37.6%) compared to the control. However, treatment with both CNP-TA and free TA reduced COX-2 levels by 34.4% and 45.3%, respectively, in irradiated cells compared to UVB control. CNPs showed no activity at the concentration tested.

## 4. Discussion

Skin photodamage originates from photon-induced changes in the skin, affecting either epidermis and dermis, attributed to continuous exposure to UV radiation, including UVA (400–315 nm) and UVB (315–280 nm) wavelengths [28]. The UVB exposure causes an imbalance between the removal of excessive ROS UVB-generated and/or an impaired ability to detoxify the reactive intermediates by the endogenous antioxidant defense system, inducing oxidative stress in skin cells [29]. Oxidative stress is the first step for photoaging and photocarcinogenesis generation. Photoaging or extrinsic aging is one of the results of photodamage, which is a consequence of exposure to outdoor agents mainly UV irradiation, overlaying the effects of natural aging (intrinsic aging) which is a constant process composed of gradual and irreversible tissue degeneration. Generally characterized by loss of elasticity, disorganized and fragmented collagen fibers, and dermal connective tissue injury [30].

The oxidative stress theory of aging is built on the structural damage-based hypothesis that the accumulation of oxidative damage to macromolecules (carbohydrates, lipids, proteins, and DNA) is associated with functional loss. This occurs probably because of senescent cells that acquire a senescence-associated secretory phenotype, including the secretion of interleukins, chemokines, and growth factors, which is involved in inflammatory events, and the secretion of degradative enzymes such as matrix metalloproteases (MMPs) [31]. Moreover, photocarcinogenesis is another consequence of photodamage, involving biochemical events that ultimately may cause skin cancer. These events are initiated by UV radiation, which is a complete carcinogen, as it initiates cancer through DNA damage via the formation of DNA photoproducts and activates oncogenes and/or silencing tumor suppressor genes, but also promotes cancer growth through inflammatory processes inherent in cumulative UV exposure [2,32].

The administration of exogenous antioxidants is a strategy used to scavenge excessive ROS and, consequently, prevent an overload of the endogenous antioxidant defense system and minimize oxidative damage in cellular components. A well-studied group of antioxidants is polyphenols, secondary metabolites of plants found largely in fruits, vegetables, cereals, and beverages. Among them, TA, a hydrolyzable tannin, has been reported to possess a variety of biological properties, including antioxidant and anti-inflammatory effects [33,34,35]. Another class of antioxidant that recently gained attention is nanoparticles that demonstrate enzyme-like activities, mimicking natural enzymes or nanozymes [36]. CNPs exhibit this property because it exists in a mixed valence state (Ce^3+^ and Ce^4+^), and due to the low redox potential of these states, allows CNPs to have self-regenerative redox cycling properties, which is evidenced as CAT and SOD-mimetic activities. Enabling them to combat excessive ROS production, as demonstrated by several studies [37,38,39].

In addition, it has been shown in many studies that the density of Ce^3+^ redox states in a given cerium oxide nanomaterial determines, in significant part, the catalytic behavior of the material towards unique ROS species. Therefore, building from these basis studies, particle formulations with controlled Ce^3+^ state densities, and thereby surface sensitivity towards ROS scavenging, may be produced by controlling the particle synthesis method. In earlier work from our group, a unique approach to synthesizing spherical particles with substantial colloidal stability and a significant presence of Ce^3+^ states in its crystal [19] was reported. In the present study, a similar synthesis was performed and subjected to a conjugation process (room temperature bioconjugation chemistry using CDI compound) with TA, aiming the investigation of the efficacy of CNP-TA in preventing photodamage in skin cells. For this, the in vitro model of L929 fibroblasts exposed to UVB-irradiation was selected, given that fibroblasts are the majority cell type comprising the dermal layer of the skin.

For CNP-TA synthesis, the activated OH group on CNPs reacted with CDI to form an active imidazole carbamate intermediate compound. Similarly, the activated OH group on TA molecules is attached to another end-amine group in the EDI molecules to form a carbamate bond. According to UV–Vis spectra results, CNP-TA nanoparticles showed peaks of TA molecule and its conjugation with CNPs. The breadth of the observed peak may arise from the secondary boning of TA molecules and their irregular aggregate structuring on the particle surface. Indicating the successful conjugation of TA on the CNP surface. From XPS results, CNP-TA decreased the ratio of Ce^3+^/Ce^4+^, compared to bare CNPs. This change is expected from the conjugation procedure, wherein the particles are put in a more alkaline condition during the conjugation and surface sites are utilized for the conjugation itself. Moreover, TA conjugation increased its size by 30% (approximately 10 nm), probably due to the association of TA molecules on the CNP surface.

For photoprotective activity, several experiments were performed to analyze the potential of CNP-TA to decrease the oxidative stress induced by UVB radiation. The 24 h treatments of either bare CNPs or CNP-TA were able to prevent the decrease in cell viability; this reflected the ability of CNP-TA to prevent the UVB-induced oxidative stress in L929 irradiated cells. In general, the induction of ROS can occur by endogenous or exogenous sources. UV radiation is the main exogenous source of ROS increase, which causes oxidative modification of skin cellular macromolecules [29]. Either TA alone, bare CNPs or CNP-TA were able to decrease ROS, this result is in line with previous findings of TA [12] and bare CNPs [13] to block ROS production in UVB-irradiated L929 fibroblasts. We found that the combination of CNPs with TA maintained the effect of diminishing ROS in the cellular environment.

To analyze the ability of the cell to cope with ROS production, it is important to analyze the endogenous antioxidant defense levels. This mechanism protects cells from free radical toxicity. The SOD and CAT are important antioxidant enzymes, SOD catalyzes the dismutation of superoxide anion (O_2_^−^) into hydrogen peroxide (H_2_O_2_), and CAT decomposes H_2_O_2_ into water and oxygen, preventing hydroxyl (OH^.^) radicals production [40]. Whereas GSH, an endogenous antioxidant agent, acts as a cofactor of several detoxifying enzymes (e.g., glutathione peroxidase), scavenges OH^.^ radicals and singlet oxygen (^1^O_2_), and regenerates the antioxidant vitamins C and E back to their active forms [41]. Here, we showed that although UVB depletes cellular levels of CAT and GSH, TA and CNP-TA treatments were able to prevent the decrease in these antioxidant defense components in UVB-irradiated fibroblasts. This result might be explained due to the ability of CNPs to act as SOD and CAT-like enzymes, preventing the depletion of endogenous antioxidant defense in cells exposed to UVB.

Additionally, the increase in ROS is related to the increase in oxidative damages to macromolecules, which leads to photodamage. Lipid peroxidation alters the cytoplasmatic membrane fluidity, leading to cell death, whereas DNA damage is among the initial steps in photocarcinogenesis and photoaging [41,42]. Here, we found that CNP-TA treatment was able to prevent UVB-induced oxidative damage in lipids and DNA molecules. The DNA fragmentation experiment using agarose gel electrophoresis can generate two different types of DNA drag patterns. If the cells are in the process of necrosis, with a non-specific DNA cleavage, the DNA pattern on the gel is a shear. However, if the cell is in the apoptosis process, with the activation of endonucleases that cleave the DNA into fragments of approximately 200-base pairs intervals, the DNA on the gel will resemble a ladder pattern [43]. In our experiment, in the UVB-irradiated group, the DNA on the gel was a continuous smear rather than a ladder, suggesting that the cells are in necrosis process. However, in the treatment groups, mainly for CNPs and CNP-TA, there was a decrease in the smear pattern, suggesting a protection against the DNA oxidative damage.

The alterations in the dermis mainly are due to collagen degradation and remodeling of the dermal extracellular matrix by MMPs, enzymes produced by keratinocytes, fibroblasts and neutrophils in response to UV radiation and inflammation [28,44]. MMP-1 is a type of metalloproteinase that directly cleaves collagen fibers, which will later be cleaved into smaller fragments by MMP-2, MMP-3 and MMP-9 [45], producing disorganization of collagen fibers in the dermis [1]. Indeed, Fisher et al. [46] reported that MMP-1 expression was increased in human UV-irradiated skin along with collagen degradation. These biological processes translate to clinical signs including wrinkle formation and reduced wound healing [30]. Here, we showed that CNP-TA treatment was able to decrease MMP-1 expression in UVB-irradiated cells. The reduction in MMP-1 was probably correlated to the improved cell proliferation at wound healing assay in L929-UVB irradiated cells treated with CNP-TA.

As inflammation is intrinsically associated with photoaging and photocarcinogenesis evolution [47], it is important to analyze inflammatory mediators that are increased in UV exposure. COX-2 is involved in arachidonic acid metabolism and mediates various inflammatory responses. COX-2 is upregulated in the skin after UV irradiation, with peak expression after 24 h [48]. There is also an age-dependent increase in UV-induced COX-2 expression that can result in chronic low-grade inflammation over time [49]. The increase in COX-2 upregulates the secretion of prostaglandin 2 (PGE2), which is a major pro-inflammatory cytokine responsible for immune cell infiltration in the skin [50]. Moreover, TGF-β is an important mediator in the acute and chronic inflammatory effects of solar radiation [51]. Chainiaux et al. [52] reported that repeated exposure to UVB induced a TGF-β-driven premature senescence in human dermal fibroblasts. We found that CNP-TA treatment was able to decrease COX-2 expression and TGF-β secretion in UVB-irradiated fibroblasts, indicating there may be a decrease in the inflammatory response.

In conclusion, this work demonstrated the synthesis of a novel delivery system for TA conjugated with CNPs and its photoprotective potential. The CNP-TA exhibited less cytotoxicity, compared to free TA, in which free TA demonstrates a cytotoxic effect in a dose-dependent manner, which was not observed with CNP-TA. In addition, the CNP-TA system showed better photoprotective activity when compared to bare CNPs, exhibiting better ROS scavenging activity, better prevention against depletion of endogenous antioxidant defense (CAT and GSH), and better prevention against oxidative damage to lipids and DNA. Furthermore, CNP-TA, improved cell proliferation, decreased MMP-1 expression, the main enzyme involved in collagen degradation, and decreased the secretion of TGF-β and COX-2 expression, suggesting the decrease in UVB-mediated inflammatory process. These results suggested that CNP-TA is a promising nanoparticulate system for preventing photodamage, minimizing the progression of photoaging and UVB-induced inflammation.

## Data Availability

Data is contained within the article.

## References

[1] El-Domyati M., Attia S., Saleh F., Brown D., Birk D.E., Gasparro F., Ahmad H., Uitto J. (2002). Intrinsic aging vs. photoaging: A comparative histopathological, immunohistochemical, and ultrastructural study of skin. Exp. Dermatol..

[2] Seebode C., Lehmann J., Emmert S. (2016). Photocarcinogenesis and Skin Cancer Prevention Strategies. Anticancer Res..

[3] Sander C.S., Chang H., Hamm F., Elsner P., Thiele J.J. (2004). Role of oxidative stress and the antioxidant network in cutaneous carcinogenesis. Int. J. Dermatol..

[4] Svobodova A., Walterova D., Vostalova J. (2006). Ultraviolet light induced alteration to the skin. Biomed. Pap. Med. Fac. Univ. Palacky Olomouc Czech Repub..

[5] Vile G.F., Tyrrell R.M. (1995). UVA radiation-induced oxidative damage to lipids and proteins in vitro and in human skin fibroblasts is dependent on iron and singlet oxygen. Free Radic. Biol. Med..

[6] Kim G.H., Kim J.E., Rhie S.J., Yoon S. (2015). The role of oxidative stress in neurodegenerative diseases. Exp. Neurobiol..

[7] Liguori I., Russo G., Curcio F., Bulli G., Aran L., Della-Morte D., Gargiulo G., Testa G., Cacciatore F., Bonaduce D. (2018). Oxidative stress, aging, and diseases. Clin. Interv. Aging.

[8] Mantena S.K., Katiyar S.K. (2006). Grape seed proanthocyanidins inhibit UV-radiation-induced oxidative stress and activation of MAPK and NF-κB signaling in human epidermal keratinocytes. Free Radic. Biol. Med..

[9] Narayanan D.L., Saladi R.N., Fox J.L. (2010). Ultraviolet radiation and skin cancer. Int. J. Dermatol..

[10] Cavinato M., Waltenberger B., Baraldo G., Grade C.V.C., Stuppner H., Jansen-Durr P. (2017). Plant extracts and natural compounds used against UVB-induced photoaging. Biogerontology.

[11] Nichols J.A., Katiyar S.K. (2010). Skin photoprotection by natural polyphenols: Anti-inflammatory, antioxidant and DNA repair mechanisms. Arch. Dermatol. Res..

[12] Daré R.G., Nakamura C.V., Ximenes V.F., Lautenschlager S.O.S. (2020). Tannic acid, a promissing anti-photoaging agent: Evidence of its antioxidant and anti-wrinkle potentials, and its ability to prevent photodamage and MMP-1 expression in L929 fibroiblasts exposed to UVB. Free. Radic. Biol. Med..

[13] Peloi K.E., Lancheros C.A.C., Nakamura C.V., Singh S., Neal C., Sakthivel T.S., Seal S., Lautenschlager S.O.S. (2020). Antioxidative photochemoprotector effects os cerium oxide nanoparticles on UVB irradiated fibroblastos cells. Colloids Surf. B Biointerfaces.

[14] Nelson B.C., Johnson M.E., Walker M.L., Riley K.R., Sims C.M. (2016). Antioxidant cerium oxide nanoparticles in biology and medicine. Antioxidants.

[15] Zholobak N.M., Ivanov V.K., Shcherbakov A.B., Shaporev A.S., Polezhaeva A.Y., Spivak N.Y., Tretyakov Y.D. (2011). UV-shielding property, photocatalytic activity and photocytotoxicity of ceria colloid solutions. J. Photoch. Photob. B Biol..

[16] Fujita N., Kamada K. (2014). Protective effect of CeO2 nanoparticles on photo-indued oxidative damage of DNA. J. Ceram. Soc. Jpn..

[17] Zholobak N.M., Shcherbakov A.B., Bogorad-Kobelska A.S., Ivanova O.S., Baranchikov A.Y., Spivak N.Y., Ivanov V.K. (2014). Panthenol-stabilized cerium dioxide nanoparticles for cosmeceutic formulations against ROS-induced and UV-induced damage. J. Photoch. Photob. B Biol..

[18] Ribeiro F.M., Oliveira M.M., Singh S., Sakthivel T.S., Neal C.J., Seal S., Ueda-Nakamura T., Lautenschlager S.O.S., Nakamura C.V. (2020). Ceria nanoparticles decrease UVA-induced fibroblasts death through cell redox regulation leading to cell survival, migration and proliferation. Front. Bioeng. Biotechnol..

[19] Neal C.J., Sakthivel T.S., Fu Y., Seal S. (2021). Aging of nanoscale cerium oxide in a peroxide environment: Its influence on the redox, surface, and dispersion character. J. Phys. Chem. C.

[20] Kolanthai E., Fu Y., Kumar U., Babu B., Venkatesan A.K., Liechty K.W., Seal S. (2022). Nanoparticle mediated RNA delivery for wound healing. Wiley Interdiscip. Rev. Nanomed. Nanobiotechnol..

[21] Borenfreund E., Puerner J.A. (1984). A simple quantitative procedure using monolayer culture for toxicity assays. J. Tissue Cult. Methods.

[22] Bradford M.M. (1976). A rapid sensitive method for the quantitation of microgram quantities of protein utilizing the principle of protein-dye binding. Anal. Biochem..

[23] Aebi H. (1984). Calase in vitro. Methods Enzymol..

[24] Hissin P.J., Hilf R. (1976). A fluorometric method for determination of oxidized and reduced glutathione in tissues. Anal. Biochem..

[25] Kasibhatla S., Amarante-Mendes G.P., Finucane D., Brunner T., Bossy-Wetzel E., Green D.R. (2006). Analysis of DNA fragmentation using agarose gel electrophoresis. Cold Spring Harb. Protoc..

[26] Bensalah N., Chair K., Bedoui A. (2018). Efficient degradation of tannic acid in water by UV/H_2_O_2_ process. Sustain. Environ. Res..

[27] Jastrzebska M., Zalewska-Rejdak J., Wrzalik R., Kocot A., Mroz I., Barwinski B., Turek A., Cwalina B. (2006). Tannic acid-stabilized pericardium tissue: IR spectroscopy, atomic force microscopy, and dielectric spectroscopy investigations. J. Biomed. Mater. Res. A.

[28] Fisher G.J., Kang S., Varani J., Bata-Csorgo Z., Wan Y., Datta S., Voorhees J.J. (2002). Mechanisms of photoaging and chronological skin aging. Arch. Dermatol..

[29] Salisbury D., Bronas U. (2015). Reactive oxygen and nitrogen species: Impact on endothelial dysfunction. Nurs. Res..

[30] Nigam Y., Knight J. (2009). Exploring the anatomy and physiology of ageing. Part 11—The skin. Nurs. Times.

[31] Pole A., Dimri M., Dimri G. (2016). Oxidative stress, cellular senescence and ageing. AIMS Mol. Sci..

[32] Black H.S., deGruijl F.R., Forbes P.D., Cleaver J.E., Ananthaswamy H.N., deFabo E.C., Ullrich S.E., Tyrrell R.M. (1997). Photocarcinogenesis: An overview. J. Photochem. Photobiol. B Biol..

[33] Andrade R.G., Ginani J.S., Lopes G.K.B., Dutra F., Alonso A., Hermes-Lima M. (2006). Tannic acid inhibits in vitro iron-dependent free radical formation. Biochimie.

[34] Erdelyi K., Kiss A., Bakondi E., Bai P., Szabo C., Gergely P., Erdodi F., Virag L. (2005). Gallotannin inhibits the expression of chemokines and inflammatory cytokines in A549 cells. Mol. Pharmacol..

[35] Chou W.-W., Wang Y.-S., Chen K.-C., Wua J.-M., Liang C.-L., Juo S.-H.H. (2012). Tannic acid suppresses ultraviolet B-induced inflammatory signaling and complement factor B on human retinal pigment epithelial cells. Cell. Immunol..

[36] Singh S. (2019). Nanomaterials exhibiting enzyme-like properties (nanozymes): Current advances and future perspectives. Front. Chem..

[37] Celardo I., Pedersen J.Z., Traversa E., Ghibelli L. (2011). Pharmacological potential of cerium oxide nanoparticles. Nanoscale.

[38] Singh S., Dosani T., Karakoti A.S., Kumar A., Seal S., Self W.T. (2011). A phosphate-dependent shift in redox state of cerium oxide nanoparticles and its effects on catalytic properties. Biomaterials.

[39] Das S., Chigurupati S., Dowding J., Munusamy P., Baer D.R., Mcginnis J.F., Mattson M.P., Self W., Seal S. (2014). Therapeutic potential of nanoceria in regenerative medicine. MRS Bull..

[40] Birben E., Sahiner U.M., Sackesen C., Erzurum S., Kalayci O. (2012). Oxidative stress and antioxidant defense. World Allergy Organ J..

[41] Valko M., Leibfritz D., Moncol J., Cronin M.T., Mazur M., Telser J. (2007). Free radicals and antioxidants in normal physiological functions and human disease. Int. J. Biochem. Cell Biol..

[42] Girotti A.W. (2001). Photosensitized oxidation of membrane lipids: Reaction pathways, cytotoxic effects, and cytoprotective mechanisms. J. Photochem. Photobiol. B.

[43] Collins J.A., Schandi C.A., Young K.K., Versely J., Willingham M.C. (1997). Major DNA fragmentation is a late event in apoptosis. J. Histochem. Cytochem..

[44] Zouboulis C.C., Makrantonaki E. (2011). Clinical aspects and molecular diagnostic of skin aging. Clin. Dermatol..

[45] Doren S.R.V. (2015). Matrix metalloproteinase interactions with collagen and elastin. Matrix Biol..

[46] Fisher G.J., Choi H.-C., Bata-Csorgo Z., Shao Y., Datta S., Wang Z.-Q., Kang S., Voorhees J.J. (2001). Ultraviolet irradiation increases matrix metalloproteinase-8 protein in human skin in vivo. J. Investig. Dermatol..

[47] Ansary T.M., Hossain R., Kamiya K., Komine M., Ohtsuki M. (2021). Inflammatory molecules associated with ultraviolet radiation-mediated skin aging. Int. J. Mol. Sci..

[48] Smith W.L., Langenbach R. (2001). Why there are two cyclooxygenase isozymes. J. Clin. Investig..

[49] Buckman S.Y., Gresham A., Hale P., Hruza G., Anast J., Masferrer J., Pentland A.P. (1998). COX-2 expression is induced by UVB exposure in human skin: Implications for the development of skin cancer. Carcinogenesis.

[50] Kim A.L., Labasi J.M., Zhu Y., Tang X., McClure K., Gabel C.A., Athar M., Bickers D.R. (2005). Role of p38 MAPK in UVB-Induced Inflammatory Responses in the Skin of SKH-1 Hairless Mice. J. Investig. Dermatol..

[51] Wang H., Kochevar I.E. (2005). Involvement of UVB-induced reactive oxygen species in TGF-β biosynthesis and activation in keratinocytes. Free. Radic. Biol. Med..

[52] Debacq-Chainiaux F., Borlon C., Pascal T., Royer V., Eliaers F., Ninane N., Carrard G., Friguet B., de Longueville F., Boffe S. (2005). Repeated exposure of human skin fibroblasts to UVB at subcytotoxic level triggers premature senescence through the TGF-β1 signaling pathway. J. Cell Sci..

